# Association between tumour infiltrating lymphocytes, histotype and clinical outcome in epithelial ovarian cancer

**DOI:** 10.1186/s12885-017-3585-x

**Published:** 2017-09-20

**Authors:** Fiona R. James, Mercedes Jiminez-Linan, Jennifer Alsop, Marie Mack, Honglin Song, James D. Brenton, Paul D. P. Pharoah, H. Raza Ali

**Affiliations:** 1 Lancashire Teaching Hospitals Foundation NHS Trust, Lancashire, UK; 2 Department of Pathology, University of Cambridge, Cambridge, UK; 3 Department of Oncology, University of Cambridge, Cambridge, UK; 4 Department of Oncology, Department of Public Health and Primary Care, University of Cambridge, Cambridge, UK; 5 Department of Pathology, CRUK Cambridge Institute, University of Cambridge, Cambridge, UK

**Keywords:** Tumour infiltrating lymphocytes (TILs), Epithelial ovarian cancer, Standardised method, Histological subtype, Survival time

## Abstract

**Background:**

There is evidence that some ovarian tumours evoke an immune response, which can be assessed by tumour infiltrating lymphocytes (TILs). To facilitate adoption of TILs as a clinical biomarker, a standardised method for their H&E visual evaluation has been validated in breast cancer.

**Methods:**

We sought to investigate the prognostic significance of TILs in a study of 953 invasive epithelial ovarian cancer tumour samples, both primary and metastatic, from 707 patients from the prospective population-based SEARCH study. TILs were analysed using a standardised method based on H&E staining producing a percentage score for stromal and intratumoral compartments. We used Cox regression to estimate hazard ratios of the association between TILs and survival.

**Results:**

The extent of stromal and intra-tumoral TILs were correlated in the primary tumours (*n* = 679, Spearman’s rank correlation = 0.60, *P* < 0.001) with a similar correlation in secondary tumours (*n* = 224, Spearman’s rank correlation = 0.62, *P* < 0.001). There was a weak correlation between stromal TIL levels in primary and secondary tumour samples (Spearman’s rank correlation = 0.29, *P* < 0.001) and intra-tumoral TIL levels in primary and secondary tumour samples (Spearman’s rank correlation = 0.19, *P* = 0.0094). The extent of stromal TILs differed between histotypes (Pearson chi2 (12d.f.) 54.1, *P* < 0.0001) with higher levels of stromal infiltration in the high-grade serous and endometriod cases. A significant association was observed for higher intratumoral TIL levels and a favourable prognosis (HR 0.74 95% CI 0.55–1.00 *p* = 0.047).

**Conclusion:**

This study is the largest collection of epithelial ovarian tumour samples evaluated for TILs. We have shown that stromal and intratumoral TIL levels are correlated and that their levels correlate with clinical variables such as tumour histological subtype. We have also shown that increased levels of both intratumoral and stromal TILs are associated with a better prognosis; however, this is only statistically significant for intratumoral TILs. This study suggests that a clinically useful immune prognostic indicator in epithelial ovarian cancer could be developed using this technique.

## Background

Ovarian carcinoma is the fourth most common cancer in women worldwide but it is responsible for the most fatalities due to gynaecological malignancy in the developed world [1]. Known prognostic factors include tumour characteristics (grade, histotype, clinical stage), patient factors (age, fitness, smoking status), treatment factors, and the response to first line treatment [2].

There is evidence that some epithelial ovarian tumours evoke an immune response and that this affects prognosis [3]. Several studies have investigated the effect of various subtypes of immune cell response on prognosis in ovarian cancer [4–20]. These studies have been of modest sample sizes ranging from 9 through to 500 tumours. Immunohistochemistry was used to investigate the immune response, but they have shown conflicting results (outlined in Table 1), though most suggest that CD3(+) and CD8(+) tumour infiltrating lymphocytes are associated with a favourable outcome. Immune infiltration has also been shown to be enriched in some molecular subtypes of ovarian cancer [21]. The anti-tumour immune response is perhaps most easily assessed morphologically using standard histopathology based on haematoxylin and eosin (H&E) staining. Therefore this may represent the most promising avenue for clinical translation. However, no study has yet investigated the effect of total tumour infiltrating lymphocytes (TILs) on prognosis in ovarian cancer.

**Table 1:** Studies investigating immune cell response and effect on prognosis in epithelial ovarian cancer

Paper	Study	Sample size	Type of immune infiltrate	Conclusion	Effect on prognosis
Woo, E.Y. et al., 2001 [4]	Immunohistochemistry and flow cytometric analysis	9	CD4(+)CD25(+) T cells CD8(+)CD25(+) T cells	Increased % of CD4(+)CD25(+) T cells present in ovarian cancer. CD8(+) T cells expressed low levels of CD25	Not discussed
Zhang et al., 2003 [5]	Immunohistochemistry	186	CD3(+) T cells	The presence of intratumoral T cells correlated with delayed recurrence and an increased expression of IFN γ, IL-2, within the tumor. The absence of intratumoral T cells was associated with increased levels of VEGF.	Increased CD3(+) T cells is associated with increased survival
Curiel, T.J., et al., 2004 [6]	Immunohistochemistry	104	CD4(+)CD25(+)FOXP3(+) T(reg) cells	T(reg) cells suppress tumor-specific T cell immunity and contribute to growth of human tumors in vivo. T(reg) cells are associated with a high death hazard and reduced survival	T(reg) cells are associated with reduced survival
Sato, E., et al., 2005 [7]	Immunohistochemistry	117	CD8(+) T cells CD3(+) T cells CD8(+)/CD4(+) T cell ratio	Increased CD8+ T cells is associated with increased survival No association between CD3+ TILs and survival Increased CD8(+):CD4(+) associated with better survival	Increased CD8+ T cells is associated with increased survival No association between CD3+ TILs and survival Increased CD8(+):CD4(+) associated with increased survival
Hamanishi, J., et al., 2007 [8]	Immunohistochemistry	70	PD-L1 expression on tumour cells CD8(+) T cells	Increased PD-L1 expression on tumour cells is associated with decreased CD8(+) T cells and decreased survival Increased CD8(+) t cells is independently associated with increased survival	Increased CD8(+) t cells is associated with increased survival
Tomsova et al., 2007	Immunohistochemistry	116	CD3(+) T cells	CD3(+) TILs are associated with increased survival	Increased CD3(+) T cells is associated with increased survival
Shah, C.A., et al., 2008 [10]	Immunohistochemistry	119	CD3(+) T cells CD8(+) T cells CD68(+) TAMs FoxP3(+) Tregs	TIL and TAM levels are positively correlated Patients with greater TILs are more likely to be optimally cytoreduced The presence of circulating tumor DNA does not correlate with TILs, TAMs, or Tregs No association between any cell type and survival seen	No association between any cell type and survival seen
Adams, S.F., et al., 2009 [11]	Immunohistochemistry	134	CD3(+) T cells CD8(+) T cells FoxP3(+) Tregs	Increased CD8(+) T cells is associated with increased survival No difference in survival associated with the other cell types	Increased CD8(+) T cells is associated with increased survival No difference in survival associated with the other cell types
Clarke, B., et al., 2009 [12]	Immunohistochemistry	500	CD8(+) T cells CD3(+) T cells	Increased CD8(+) T cells is associated with increased survival Increased CD3(+) T cells is not associated with any difference in survival On subgroup analysis: For serous ovarian carcinomas, increased CD3(+) and CD8(+) T-cells correlated with improved survival For endometrioid and clear cell carcinomas there is no association between CD3(+) or CD8(+) and survival	Increased CD8(+) T cells is associated with increased survival Increased CD3(+) T cells is not associated with any difference in survival
Leffers et al., 2009 [13]	Immunohistochemistry	306	CD8(+) T cells CD45RO(+) Tmems FoxP3(+) Tregs	increased CD8(+) CTL and CD8(+)/FoxP3(+) ratio is associated with increased survival in advanced stage patients increased CD8(+) T cells and FoxP3(+) Tregs is associated with increased survival	increased CD8(+) CTL and CD8(+)/FoxP3(+) ratio is associated with increased survival in advanced stage patients increased CD8(+) T cells and FoxP3(+) Tregs is associated with increased survival
Stumpf et al., 2009 [14]	Immunohistochemistry	100	CD20(+) B cells CD3(+) T cells CD4(+) T cells CD8(+) T cells	CD3(+) T cells and CD8(+) T cells are associated with increased survival	CD3(+) T cells and CD8(+) T cells are associated with increased survival
Webb et al., 2014 [15]	Immunohistochemistry	497	CD103(+) T cells	CD103(+) TILs comprise intraepithelial, activated CD8(+) T cells, and NK cells and are strongly associated with patient survival in HGSC	CD103(+) TILs are strongly associated with patient survival in HGSC
Darb-Esfahani et al. 2016 [16]	Immunohistochemistry	215	CD3+, PD-1+, and PD-L1+ T cells	CD3+, PD-1+, and PD-L1+ TILs densities were correlated with increased survival Moreover, high PD-1+ TILs as well as PD-L1+ TILs densities added prognostic value to CD3 + TILs	CD3+, PD-1+, and PD-L1+ TILs densities were correlated with increased survival in HGSC
Woulters et al. 2016 [17]	Immunohistochemistry	171	CD8(+) T cells CD27(+) T cells	A prognostic benefit for patients with high intratumoral CD8(+) TIL was observed if primary surgery had resulted in a complete cytoreduction, optimal or incomplete cytoreduction fully abrogated the prognostic effect of CD8(+) TIL. Neither CD8(+) nor CD27(+) cell infiltration was of prognostic benefit in patients treated with neoadjuvant chemotherapy.	CD8+ TILs interact with treatment to affect prognosis
Bösmüller et al. 2016 [18]	Immunohistochemistry	135	CD3 T cells CD103 T cells	Both the presence of CD103 cells, as well as high numbers of intraepithelial CD3 lymphocytes (CD3E), showed a significant correlation with overall survival	Both the presence of CD103 cells, as well as high numbers of intraepithelial CD3 lymphocytes (CD3E), showed a significant correlation with overall survival
Strickland et al. 2016 [19]	Immunohistochemistry	245	CD3+ T cells CD8+ T cells	Increased CD3+ and CD8+ TILs associated with increased survival	Increased CD3+ and CD8+ TILs associated with increased survival
Kroeger et al. 2016 [20]	immunohistochemistry		CD20+ Tcells CD4+ Tcells CD8+ T cells Plasma cells	CD8(+) TIL carried prognostic benefit only in the presence of PCs and these other TIL subsets.	CD8(+) TIL carried prognostic benefit only in the presence of PCs and these other TIL subsets.

Table abbreviations: *Tregs* regulatory T cells, *TAM* tissue associated macrophages, *Tmems* memory T cells

There have been calls for the use of a standardised method of evaluating TILs in cancer and incorporating it clinically as a prognostic variable [22]. Haematoxylin and eosin (H&E) staining and visual analysis is a recognised cheap and clinically accessible method of evaluating TILs. A standardised method for H&E visual evaluation of TILs has been validated in the context of breast cancer [23].

We investigated the pathological and prognostic significance of TILs in epithelial ovarian cancer. We used a standardised method of TIL evaluation and a large prospective population-based study of epithelial ovarian cancer to achieve this.

## Methods

### Study population

The SEARCH ovarian cancer study is an ongoing, population-based ovarian cancer case–control study, covering the regions served by the East Anglia and West Midlands cancer registries in the UK. All patients diagnosed in East Anglia with invasive epithelial ovarian cancer under the age of 70 years since 1991, and still alive in 1998 when recruitment started are eligible to participate. Details of this study and its participants have been published previously [24]. To date over 2500 women with epithelial ovarian cancer have been recruited. We have retrieved archival, formalin-fixed, paraffin-embedded (FFPE) tumour tissue from the primary surgery from 707 SEARCH participants with invasive epithelial ovarian cancer. A total of 953 samples have been retrieved including tumour from both primary and metastatic sites.

Survival time data were available through the regional cancer registry which obtains notification of deaths by death certificate flagging through the Office of National Statistics. The lag times for this are a few weeks for cancer deaths and two months to a year for non-cancer deaths. In addition, the cancer registry checks vital status by querying the National Health Service Strategic Tracing Service. Vital status was ascertained at the end of June 2015 and all analyses were censored on 31 December 2014 to allow for delay in reporting of vital status. Ovarian cancer specific mortality was defined as deaths where ovarian cancer was listed as the cause of death on Parts 1a, 1b, or 1c of the death certificate.

### Evaluation of tumour infiltrating lymphocytes

Formalin-fixed, paraffin-embedded tumour samples were sectioned and stained using hematoxylin and eosin. The morphology was reviewed by a gynaecological pathologist (MJ-L). Full face stained sections were then scanned and the digital images were used for evaluation of TILs.

**Fig. 1:**
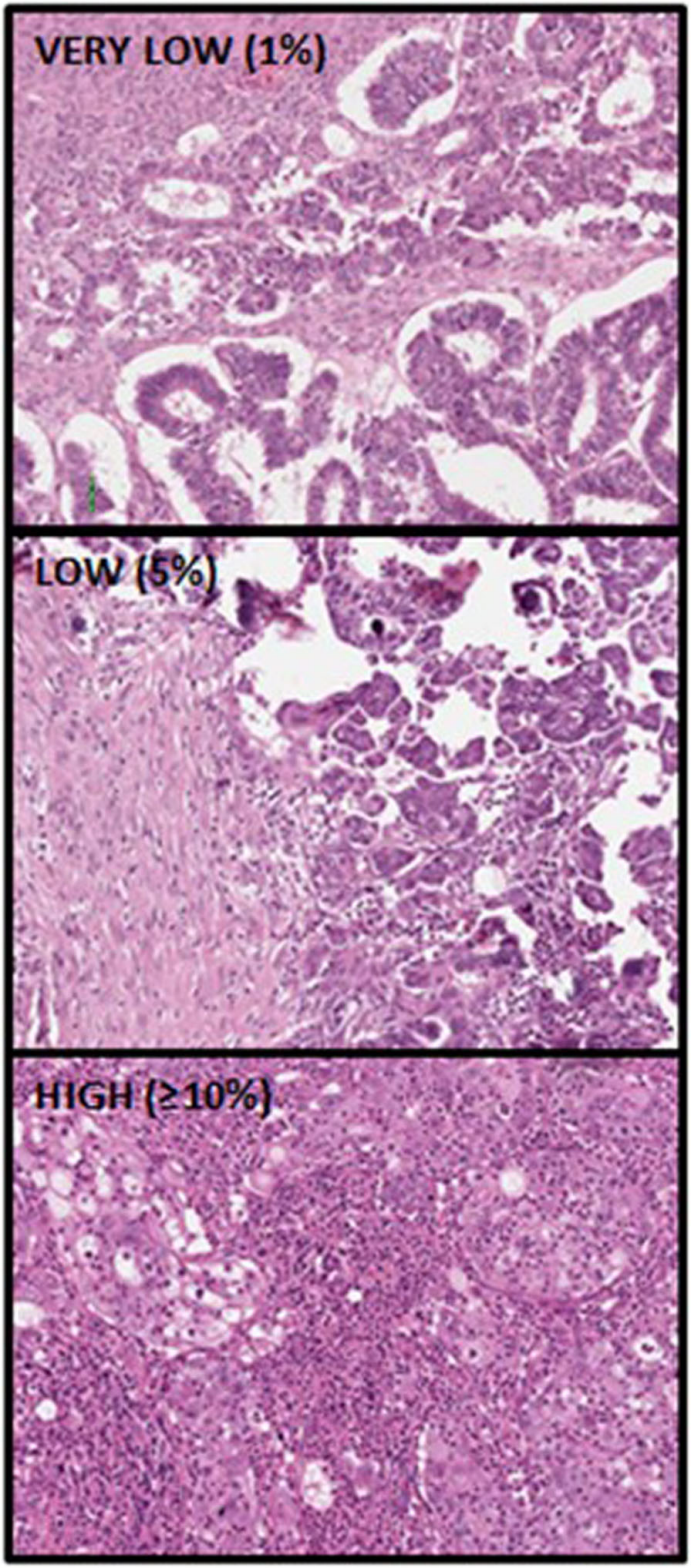
Representative examples of tumours with very low, low and high TIL

Evaluation of TILs was performed by manual visual assessment of percentage area covered by lymphocytes, following a standardised method as described in Salgado et al. [23]. In this method the area within the tumour border is selected, the intra-tumoral and stromal compartment are defined, only areas of mononuclear lymphocytic infiltrate are included, and the percentage area is assessed at low (4× objective) and high magnification (10× objective). The percentage categories used were 1%, 5% and the nearest 10% up to 100%. The intra-tumoral and stromal compartments were scored separately. The intra-tumoral and stromal TILs were classified into three groups for the purpose of analysis – very low (1%), low (5%) and high (≥10%). Representative examples of tumours with very low, low and high TIL are shown in Fig. 1.

### Statistical analysis

The proportion of tumours in the three TIL score categories by other histopathologic characteristics was compared using the Pearson χ2- test. Multi-variable Cox regression was used to assess the association between TILs and disease-specific mortality. Follow-up time was adjusted for delayed study entry by using the date of study entry as the start of time at risk (left truncation) [25]. All models were adjusted for FIGO stage (stage 1–4 treated as an ordinal variable), histotype (seven categories) and age at diagnosis. Analyses were censored at 10-years of follow-up or on death from a cause other than ovarian cancer. Stata 14 (Stata Corp, College Station, Texas, USA) statistical software was used for all analyses.

## Results

**Fig. 2:**
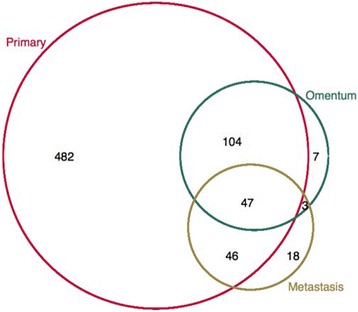
Number of patients by available tumour sample

Data were available for 953 invasive epithelial ovarian tumours (primary and/or metastatic), from 707 women. Of these, primary tumour material was available for 682 cases, omental tumour material for 161 cases and other metastasis material was available for 114 cases (Fig. 2). The clinical characteristics (age at diagnosis and stage) of these cases by histotype are shown in Table 2.

**Table 2:** Clinical characteristics of ovarian cancer cases included in this study

Histotype	Mean age at diagnosis	Stage			
		I/II (%^a^)	III/IV (%)	Unknown	Total
LGSC	54.0	5 (25)	15 (75)	3	23
HGSC	57.5	80 (29)	192 (71)	42	314
Mucinous	52.7	39 (91)	4 (9)	5	48
Endometrioid	54.5	113 (91)	11 (9)	11	135
Clear cell	55.7	70 (91)	7 (9)	5	82
Other	56.3	39 (64)	22 (36)	8	69
Borderline	49.6	33 (100)	0 (0)	3	36
Total	55.7	379 (60)	251 (40)	77	707

*LGSC* low-grade serous cancer, *HGSC* high-grade serous cancer

^a^percent of those with known stage

There was a moderately strong correlation between the extent of stromal and intra-tumoral TIL levels in the primary tumour (*n* = 679, Spearman’s rank correlation = 0.60, *P* < 0.001) with a similar correlation in secondary tumours (*n* = 224, Spearman’s rank correlation = 0.62, *P* < 0.001). Secondary tumour samples were either omental metastases or metastases at other sites. We used the data for the omental metastases in preference to data for other metastases if present; otherwise the data from the other metastases was used. We also evaluated the correlation between TIL levels in primary and metastatic tumours using data from the 196 women for whom both a primary and a secondary tumour sample was available. There was a statistically significant but weak correlation between stromal TIL levels in primary and secondary tumour samples (Spearman’s rank correlation = 0.29, *P* < 0.001) and intra-tumoral TIL levels in primary and secondary tumour samples (Spearman’s rank correlation = 0.19, *P* = 0.0094) (Table 3 *)*.

**Table 3:** Association between tumour infiltrating lymphocyte levels in primary and secondary tumours for stromal and intratumoral infiltrating lymphocytes

Primary tumour infiltrating lymphocytes	Secondary tumour infiltrating lymphocytes			
	<5%	5–9%	≥10%	Total
*Stromal*				
< 5%	21	25	39	85
5–9%	7	17	39	63
≥10%	4	8	37	49
Total	32	50	115	197
*Intra-tumoral*				
< 5%	96	55	19	170
5–9%	6	4	5	15
≥10%	3	5	3	11
Total	105	64	27	196

The extent of stromal TIL levels differed between histotypes (Table 4, Pearson chi2 (12d.f.) 54.1, *P* < 0.0001) with higher levels of stromal infiltration in the high-grade serous and endometriod cases. However, there was little difference in the degree of intra-tumoral infiltrating lymphocytes by histotype (Pearson chi2 (12d.f.) = 15.0, *P* = 0.24).

**Table 4:** Intra-tumoral and stromal tumour infiltrating lymphocyte levels by histotype

Histotype	Tumour infiltrating lymphocyte level Number (%)			
	<5%	5–9%	≥10%	Total
*Stromal*				
Low-grade serous	13 (57)	8 (35)	2 (9)	23
High-grade serous	122 (39)	85 (27)	107 (34)	314
Mucinous	28 (58)	12 (25)	8 (17)	48
Endometrioid	68 (50)	29 (22)	38 (28)	135
Clear cell	58 (71)	12 (15)	12 (14)	82
Other	33 (48)	18 (26)	18 (26)	69
Borderline	30 (83)	4 (11)	2 (6)	36
Total	352 (50)	168 (24)	187 (26)	707
*Intra-tumoral*				
Low-grade serous	20 (87)	1 (4)	2 (9)	23
High-grade serous	240 (76)	47 (15)	27 (9)	314
Mucinous	43 (90)	3 (6)	2 (4)	48
Endometrioid	108 (80)	20 (15)	7 (5)	135
Clear cell	69 (84)	8 (10)	5 (6)	82
Other	58 (84)	8 (12)	3 (4)	69
Borderline	34 (94)	2 (6)	0 (0)	36
Total	572 (81)	89 (13)	46 (7)	707

We assessed the impact of tumour infiltrating lymphocytes on ovarian cancer specific mortality after diagnosis using multi-variable Cox proportional hazards regression. The extent of tumour infiltrating lymphocytes in three categories (<5%, 5–9%, ≥10%) was treated as an ordinal variable in the model. An increase in stromal TIL levels was associated with a slightly better prognosis but the difference was not statistically significant (HR 0.87 95% CI 0.74–1.02 *p* = 0.091). A slightly stronger and nominally significant association was observed for intra-tumoral TIL levels (hazard ratio = 0.74, *P* = 0.047). When the extent of both stromal and intra-tumoral TIL levels were included in a multi-variable model the effect of the intra-tumoral TIL levels was similar (HR 0.76 95% CI 0.58–1.00 *p* = 0.047), but the association of the stromal lymphocytes was attenuated (HR 0.97 95% CI 0.79–1.19 *p* = 0.78). There was little evidence for heterogeneity of effect of intra-tumoral TIL levels by histotype, although the effect was strongest in the high-grade serous cases (HR 0.70 95% CI 0.50–0.97 *p* = 0.034).

## Discussion

This study is the largest collection of epithelial ovarian tumour samples evaluated for TILs, with almost double the number of tumour samples as the largest published study to date [12]. This is also the first time this standardised H&E visual assessment method of TIL evaluation has been used in ovarian tumours.

We have shown that TILs as assessed by stromal lymphocyte levels correlate with clinical variables such as tumour histological subtype. However, intratumoral TILs did not. We have also shown that increased levels of both intratumoral and stromal TILs are associated with a better prognosis; however, this is only statistically significant for intratumoral TILs.

These results add weight to the argument that the immune system plays an important role in ovarian cancer. They also support the idea that different subtypes of ovarian cancer are variably immunogenic and that the immune response can help define different subtypes.

The difference between stromal and intratumoral TIL effects, despite their correlated levels in both primary and secondary tumours, is interesting, and warrants further investigation. The greater prognostic effect of intratumoral TILs may explained by the biological rationale that T-cell activation necessitates physical contact with target cells in order to engage the T cell receptor [26]. In contrast, the lesser prognostic effect of stromal TILs might be explained in two ways; either they are in-transit to tumour cells hence will eventually become intratumoral TILs, or that they have been ‘excluded’ from the tumour microenvironment by factors secreted by tumour cells and/or other stromal cells, as a means of evading immune attack [26]. Further work to elucidate the distinct roles of intratumoral and stromal TILs is needed.

The results suggest that a clinically useful immune prognostic indicator in epithelial ovarian cancer could be developed using this technique, but larger scale studies are needed to replicate these results. Thus, further studies evaluating the potential of TILs as predictors of treatment response are warranted.

## Conclusion

This study is the largest collection of epithelial ovarian tumour samples evaluated for TILs. We have shown that stromal and intratumoral TIL levels are correlated and that their levels correlate with clinical variables such as tumour histological subtype. We have also shown that increased levels of both intratumoral and stromal TILs are associated with a better prognosis; however, this is only statistically significant for intratumoral TILs. This study suggests that a clinically useful immune prognostic indicator in epithelial ovarian cancer could be developed using this technique.

## Abbreviations

H&E: Hematoxylin and eosin
ITL: Intratumoral lymphocytes
SL: Stromal lymphocytes
TILs: Tumour infiltrating lymphocytes
